# Antimicrobial Activity of Three Root Canal Irrigants on Enterococcus Faecalis: An in Vitro Study

**Published:** 2008-04-02

**Authors:** Zohreh Ahangari, Mohammad Samiee, Mohammad Amin Yolmeh, Gita Eslami

**Affiliations:** 1 *Department of Endodontics, Dental School, Shahid Beheshti University of Medical Sciences, and Member of Iranian Center for Endodontic Research, Tehran, Iran*; 2 *Endodontist, Tehran, Iran*; 3 *Department of Microbiology, Medical School, Shahid Beheshti University of Medical Sciences, Tehran, Iran*

**Keywords:** Antimicrobial, Endodontics, Enterococcus Faecalis, Irrigation, Sodium Hypochlorite

## Abstract

**INTRODUCTION:** The aim of this study was to compare the antimicrobial effects of 2.5% Sodium hypochlorite (NaOCl), 2% Chlorhexidine Gluconate (CHX) and BioPure MTAD (MTAD) on Enterococcus (E) *faecalis*-contaminated root canals of human extracted teeth.

**MATERIALS AND METHODS:** Seventy human intact extracted single-rooted teeth with straight root canal randomly divided into 5 groups: positive control (n=5), negative control (n=5), 2.5% NaOCl (n=20), 2% CHX (n=20), and MTAD (n=20). Each tooth was instrumented using the passive step-back technique hand and rotary instruments. *E. faecalis *incubated into the canals and grew for 4 weeks. Canals irrigated using three mentioned solutions for 5 minutes. Samples were taken from canal walls and transferred into Brain Heart Infusion (BHI) culture medium and placed in an incubator at 37ºC for 96 hours and bacteriological evaluations were done. Chi- Square test and SPSS software were used for the statistical analysis of the results.

**RESULTS:** Bacterial growth was seen in only one sample of MTAD group (5%), but in 4 of CHX group (20%) and 5 of NaOCl group (25%). Chi-Square test showed no statistically difference between groups.

**CONCLUSION:** Based on the results of this study, it seems that all three solutions have acceptable antimicrobial effect on *E. faecalis.*

## INTRODUCTION

One of the most important reasons of endodontic treatment failure is the persistence or survival of microorganisms in the complex root canal system or periapical area; therefore, the success of endodontic treatment depends, to a great degree, on the elimination of microorganisms from the root canal system (1). However, irrigating the canals with antimicrobial solutions is an important step to decrease the number of microorganisms or eliminate them from the root canal system; therefore, appropriate irrigating solutions for this stage of the treatment should be evaluated and introduced.

According to Sundquist the presence of E. faecalis in the root canal system of 38% of teeth with endodontic treatment failure was demonstrated. Furthermore, only 33% of the teeth which harbored *E. faecalis *when the canals were being refilled had demonstrated endodontic success. Therefore, *E. faecalis *is an important factor in endodontic treatment failure and its presence at the time of canal filling lowers the rate of treatment success to great extent (2).

*E. faecalis *is completely resistant to intra-canal medications and is one of the microorganisms which resist against antimicrobial properties of calcium hydroxide (2) and is able to survive in root canals without synergistic effect of other bacteria (3).

Different kinds of antimicrobial rinses have been introduced for disinfecting the root canal system; all these solutions have disadvantages such as limited antimicrobial activity, non-selectivity for host cells, inability to penetrate into dentinal tubules and a risk of allergy and toxicity in patients. Therefore, no ideal intra-canal medication is available (4).

Sodium hypochlorite (NaOCl) has long been the irrigant of choice for nonsurgical endodontic procedures (5). NaOCl dissolves necrotic and vital tissue, has antimicrobial activity, and aids as a lubricant in canal (5). However, NaOCl is extremely toxic to the periapical tissues if it is passed beyond the tooth apex (6). Chlorhexidine gluconate (CHX) has been shown to be a broad-spectrum antimicrobial agent that has the advantage of substantivity (7). CHX has also been shown to be more effective against gram-positive organisms than gram-negative organisms (8).

BioPure MTAD (Mixture of doxycycline, citric acid, and Tween 80) has been suggested as a final rinse because of its antimicrobial properties and its ability to remove the smear layer. BioPure MTAD (MTAD) is less cytotoxic than 5.25% NaOCl (9-10).

The purpose of this in vitro study was to compare the antimicrobial effects of 2.5% NaOCl, 2% CHX and MTAD as root canal irrigant on *E. faecalis*-contaminated root canals of extracted human teeth.

## MATERIALS AND METHODS

Seventy sound single-rooted human teeth which had been extracted because of periodontal diseases or orthodontic treatment were selected. The teeth included maxillary and mandibular incisors and mandibular premolars and had one canal each only.

The external surfaces of the roots were immediately debrided after extraction and all the adherent tissues were removed using a curette. Then all teeth were placed in 0.5% NaOCl for 24 hours for surface disinfection and were stored in 0.9% sterile physiologic serum at room temperature until used.

In the first step of the study, the crowns of all teeth were cut away at CEJ perpendicular to the long axis of teeth using a long cylindrical bur in a high speed handpiece, under copious amounts of water spray. The remaining roots measured 12-18 mm. Then #10 and #15 K-files (Mani, Japan) were used to make sure that the roots had only one canal and the canal was patent. All canals were prepared in a similar manner to decrease the number of confounding variables. In the first step, an appropriate-sized Hedström file (Maillefer, Ballaigues, Switzerland) was used to remove pulp remnants and debris from the canals. Then a #15 or #20 file was inserted into the canals so that the tip of the file was visible at the apical foramen. Then the working length was determined 1 mm short of the file penetration into the canal.

Passive step-back method was used to prepare the canals using GG drills, hand files and Flex- Master rotary files (Maillefer, Ballaigues, Switzerland). All canals were instrumented with #35 hand K-files as master apical files (MAF). The canals were recapitulated and irrigated with 0.5% NaOCl solution between filings. Debridement and shaping of the canals were completed using 5 mL of sterile normal saline.

Subsequent to canal preparation, the apical foramina of all the specimens were sealed with cyanoacrylate glue to prevent bacterial microleakage (10-11). All the specimens were placed in an ultrasonic container (Ultrasonic Bath, Vector 55, Jeltraft, Jelenko) to remove the smear layer completely. The specimens were placed in 17% EDTA for 10 minutes, followed by 5.25% sodium hypochlorite for 10 minutes. The specimens were finally immersed in sterile distilled water for 10 minutes (12).

Specimens were placed in capped laboratory containers containing BHI (Brain Heart Infusion) broth and put in an autoclave at 121ºC and 15 PSI for 20 minutes for sterilization. Subsequent to sterilization all the specimens were transported and manipulated under aseptic conditions using sterile instruments and equipment. (13).

To induce controlled and standard infections pure *E. faecalis *suspensions (ATCC 29212) were injected into the canals using sterile insulin syringes under aseptic conditions. The canals were completely filled with the suspension and the specimens were separately placed in special flasks containing 2 mL of sterile BHI broth (14). Five specimens did not receive suspension injections and were kept intact to serve as negative controls to confirm sterilization and the reliability of the procedures in the laboratory as to the absence of accidental microbial contamination.

In the next step, all specimens were kept in special flasks in an incubator at 37ºC for 4 weeks. All the specimens were monitored during this period (15). Subsequent to incubation, all the specimens were separately retrieved from the flasks under aseptic conditions. Then each specimen was immersed in 3 mL of sterile physiologic serum in a test tube and was shaken 3 times for 30 seconds each time on a rotator to remove the excess of the culture media from the specimens. In addition, a large number of the bacteria present on the external surfaces of the specimens were removed during irrigation and rinsing (16). SEM evaluation of two samples demonstrated sufficient penetration of microorganisms into dentinal tubules.

Subsequently, test irrigating solutions were used as follows: In the first group, 1 mL of BioPure MTAD (Dentsply Tulsa Dental, Johnson City, USA) solution was injected into contaminated specimens using special intra-canal syringes. Then each specimen was placed in special flasks containing 2 mL of MTAD for 5 minutes (13). In the specimens in groups 2 and 3, 1 mL of 2% CHX (Shahredaroo, Tehran, Iran) and 1 mL of 2.5% NaOCl (Pakshoma, Tehran, Iran) were, respectively, injected into the canals using insulin syringes and the specimens were immersed in 2 mL of the above-mentioned solutions in special flasks for 5 minutes. In the positive control group, the specimens were placed in contact with sterile physiologic serum in the same manner. Negative control specimens which had not been inoculated with any microorganisms and were sterile were not exposed to any solutions. Subsequent to the removal of the specimens from the flasks each specimen was rinsed three times with 3 mL of sterile physiologic serum on a rotator to remove all irrigating solution from the surface of the specimens. Samples were taken from the specimens in the three experimental groups and the negative and positive control groups separately using #40 Hedström files in aseptic conditions. The samples were transported into test tubes containing BHI and placed in an incubator at 37ºC for 96 hours (13).

The samples were retrieved from the incubator after 96 hours to be evaluated bacteriologically as to the presence of microorganisms, their exact number in the liquid culture media and the observation of colonies formed. Clear test tubes demonstrated complete sterilization and blurred test tubes were considered contaminated samples. Samples were taken from the contaminated specimens using standard 0.001 aluminum loops and cultured in Bile-Esculin Agar (BEA), which is a specific culture medium for *E. faecalis *(16).

**Table 1:** Frequency distribution of bacterial growth in samples from different groups

	*NaOCL*	*CHX*	*MTAD*	*Saline*
**No growth**	15(75)^a^	16(80)	19(95)	0(0)
**Growth**	5(25)	4(20)	1(5)	5(100)

***a:*** *Number (Percent)*

The plates were placed in a CO_2_ incubator for 24 hours. Then the plates were evaluated for the presence of bacteria and the constituents of the colonies.

Chi-square test and SPSS Software were used for the statistical analysis of the results. Quantitative analysis was not performed since the colony counts were 10^5^ CFU/mL or higher in all the cases.

## RESULTS

All 5 samples from the positive control group demonstrated bacterial growth. However, none of the samples from the negative control groups manifested bacterial growth. In the MTAD group in one sample (5%) bacterial growth was observed. Bacterial growth was present in 5 samples (25%) in the 2.5% NaOCl group and in 5 samples (20%) in the CHX group, demonstrating no statistically significant differences. In all the bacterial growth cases, colony count was 105 CFU/mL or higher. Table 1 summarizes the results of the present study.

## DISCUSSION

An ideal intra-canal irrigating solution should be able to disinfect the dentin and dentinal tubules in the first treatment session and maintain its antimicrobial potential for some time after being used (17).

*E. faecalis *was selected for the purpose of the present study because it is believed that it is one of the intra-canal bacteria which are most resistant to elimination by disinfecting agents (18). Flaring the canals with #35 K-file helped the irrigating solutions better penetrate into the canals so that lack of penetration of the solutions into the apical third of the canals would not be interpreted as lack of antimicrobial activity.

The duration of exposure to irrigating solutions was selected on the basis of studies by Gomes *et al., *Torabinejad *et al., *and Shabahang and Torabinejad (10,17,19). An interval of 4 weeks for bacterial growth was selected based on the study carried out by Shabahang and Torabinejad (10).

The samples from the canal walls were taken using Hedström files since the samples were meant to be taken from dentinal tubules and this process can also evaluate the penetration of irrigating solutions into the dentinal tubules. NaOCl is a commonly used intra-canal irrigating solution and its antibacterial properties are attributed to hypochlorous acid (20). Sjögren *et al. *demonstrated that approximately 40% of the canals remain contaminated subsequent to debridement with 2.5% NaOCl (21). According to a study by Shuping *et al. *up to 30% of the canals are contaminated after being irrigated with 1.25% NaOCl (22). Siqueira *et al. *demonstrated that the rate of canal contamination subsequent to the use of NaOCl is 30-40% (23).

Chlorhexidine is a broad-spectrum cationic bis- guanide with antimicrobial effects on gram- positive and gram-negative bacteria (12, 20). Contrary to NaOCl, CHX (with a concentration of 2% in the gel and liquid forms) preserves its antimicrobial effect for some time after being used (residual effect or substantivity), but it is unable to dissolve tissues (10, 12, 24). According to Schafer and Bossman, 2% CHX is more effective compared to its lower concentrations, manifesting its influence in a shorter period of time and with a proper antimicrobial influence on *E. faecalis *(25). Therefore, 2% CHX was used in the present study. Ercan *et al. *demonstrated that 2% CHX inhibits *E. faecalis *in 80% of the cases, which is consistent with our results.

MTAD is a new irrigating solution compared to the other two solutions tested in the present study. It removes the smear layer with less erosion in comparison with EDTA (9, 27), and contrary to EDTA, destroys *E. faecalis *in 2-5 minutes.

MTAD preserves its antibacterial properties even after being diluted 200 times, whereas antibacterial activity of NaOCl is maintained up to 32 times of being diluted (17). Doxycycline present in MTAD can remove organic and non- organic material from the root surface and preserves its effects for a long time since it is chelated to calcium ions. Doxycycline has extensive activity in the presence of citric acid and Tween 80 (polysorbate), which is a detergent and lowers surface tension (13). Low pH (lower than 3), anti-collagenase activity and a dentin- binding ability, resulting in its slow release, are prominent properties of doxycycline (10).

Torabinejad *et al. *did not demonstrate any differences in the antimicrobial influence of MTAD and 5.25 NaOCl on *E. faecalis *(17). Portenier *et al. *demonstrated that MTAD and 2% CHX do not differ in their ability to destroy *E faecalis *(28). In the present study, MTAD had not inhibited *E. faecalis *only in one sample (a 95% success rate); however, no statistically significant differences were observed among these solutions.

## CONCLUSION

Based on the results of this study, it seems that all three solutions have acceptable antimicrobial effect on *E. faecalis *in extracted human teeth. Further studies are needed to determine the effect of these findings in clinical settings.
